# The 27th European conference on biomaterials: facts and figures

**DOI:** 10.1007/s10856-016-5702-9

**Published:** 2016-03-16

**Authors:** Elżbieta Pamuła, Jan Chłopek

**Affiliations:** Department of Biomaterials, Faculty of Materials Science and Ceramics, AGH University of Science and Technology, Al. A Mickiewicza 30, 30-059 Kraków, Poland

The Polish Society for Biomaterials (PSB) and AGH University of Science and Technology (AGH) hosted the 27th Conference of the European Society for Biomaterials (ESB). The conference was held in Kraków, Poland from 30th August to 3rd September 2015 at the brand new ICE Congress Center.

**Fig. 1:**
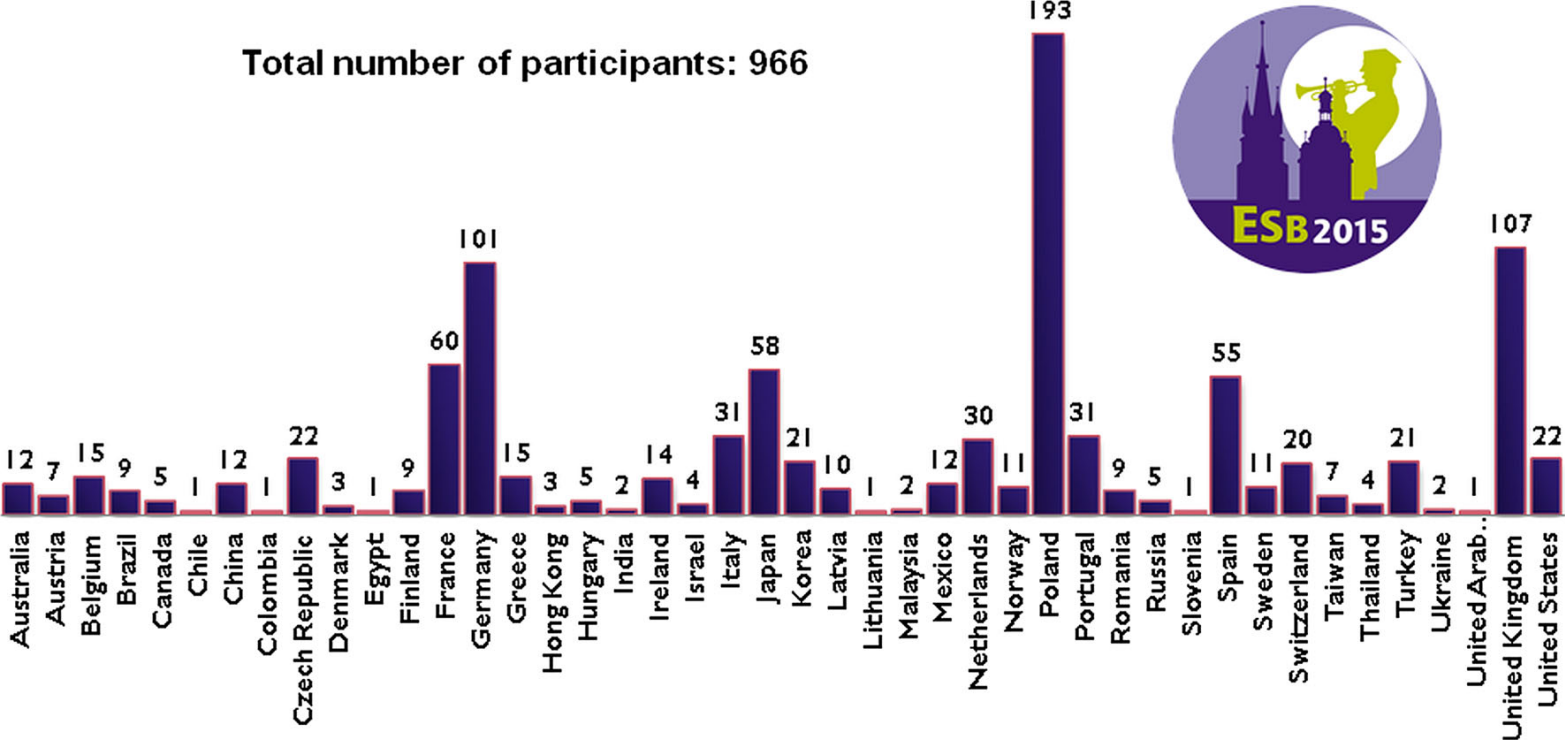
Attendance per country at ESB 2015

More than 966 participants from 44 countries attended the ESB 2015 conference. Figure 1 shows the number of attendees from different countries: the highest number of participants came from Poland, United Kingdom, Germany, France, Japan, Spain, Italy, Portugal and the Netherlands. The participants were mostly European, although there were also a considerable number of attendees from overseas countries such as Japan, US, Korea, Australia, China, Mexico and many others.

**Fig. 2:**
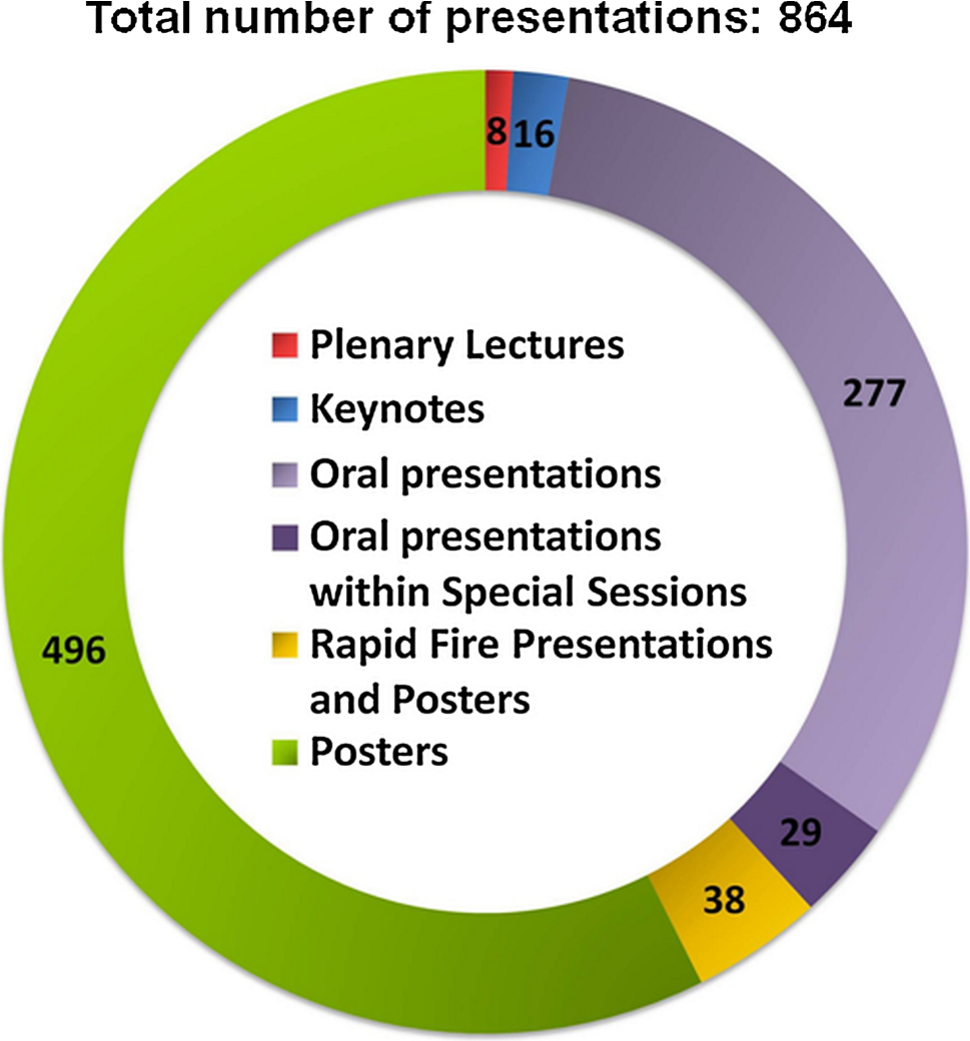
Presentation numbers and types at ESB 2015

The scientific program included 58 Sessions and was comprised of 277 oral presentations, 38 rapid fire and 534 poster presentations (Fig. 2). The program contained eight plenary lectures given by: Prof. Joachim Kohn of the New Jersey Center of Biomaterials, US; Prof. Maria Siemionow of University of Illinois at Chicago, US; Prof. C. James Kirkpatrick of University Medical Center Johannes Gutenberg University of Mainz, Germany; Prof. Geoff Richards of AO Research Institute Davos, Switzerland; Prof Michael V. Sefton of University of Toronto, Canada; Prof. Kazunori Kataoka of University of Tokyo, Japan; Prof. Małgorzata Lewandowska-Szumieł of Medical University of Warsaw, Poland and Prof. Abhay Pandit of National University of Ireland. There were also 16 keynote lectures presented by renowned international experts and 2 lectures by award winners: the International Award for Prof. Nicholas A. Peppas and the Jean Leray Award for Dr. Joachim M. Oliveira. In addition we were pleased to host special sessions, e.g. the Young Scientist Forum, the Special Fellows Session, the Translational Research Symposium and Science for Industry, during which 29 additional presentations were given. During ESB 2015 more than 24 companies were present in the exhibition area.

**Fig. 3:**
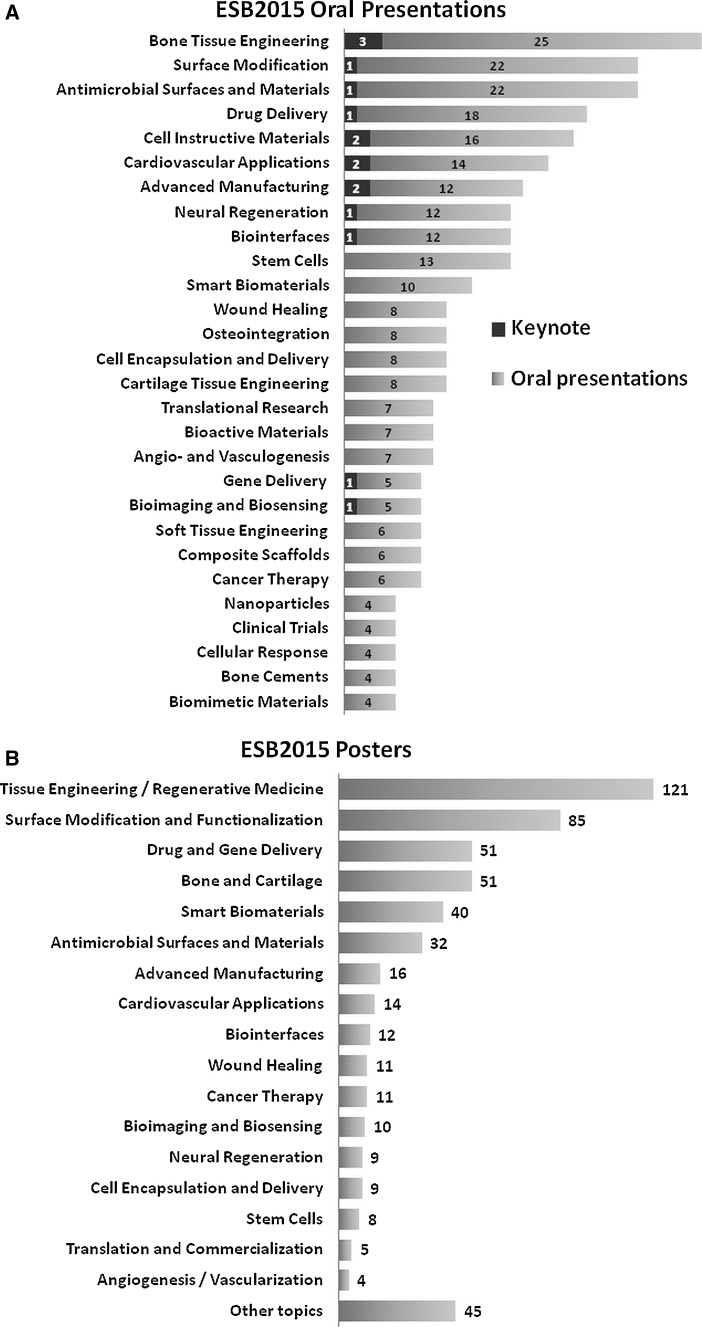
Number of oral **a** and poster **b** presentations delivered during ESB 2015 assigned to different topic sessions

The main motto of the 27th ESB was “*Balancing the needs for basic research and translation in a time of limited resources*”. To address this issue, the conference program was designed to cover a variety of topics, including recent advances in tissue engineering, surface modification, advanced manufacturing, cell instructive materials, antimicrobial surfaces and materials, biointerfaces, drug and gene delivery systems, stem cells, and many more. Figure 3 shows the number of oral (A) and poster (B) presentations delivered during ESB 2015 presented in particular topic sessions.

An exceptional attendance of young scientists was noted at ESB 2015: 376 students were registered and 333 applied for the ESB best oral/poster awards. In total, young scientists submitted over 500 out of a total of 912 abstracts. The Young Scientists Forum (YSF) workshop devoted to the topic “*From Creative Thinking Towards Product Commercialization: The Complete Value Chain*” was greatly appreciated. The YSF board members were chairing and evaluating rapid fire presentations. The young scientists were also invited to co-chair the sessions. This year a Special Fellows Session, organized by the International College of Fellows of Biomaterials Science and Engineering (IUS-BSE) and chaired by Prof. Joachim Kohn, was dedicated to biomaterials education and entitled “*Biomaterials Education is not ready for the Challenges of the Future*”. A group of six prominent Fellows highlighted the perceived strengths and weaknesses of the way the universities educate and train the next generation of biomaterials scientists and prepare them for their careers.

The ESB 2015 allowed the participants to present their latest results as well as to share their knowledge and expertise and to discuss their visions of the future of biomaterials science and biomaterials community.

**Fig. 4:**
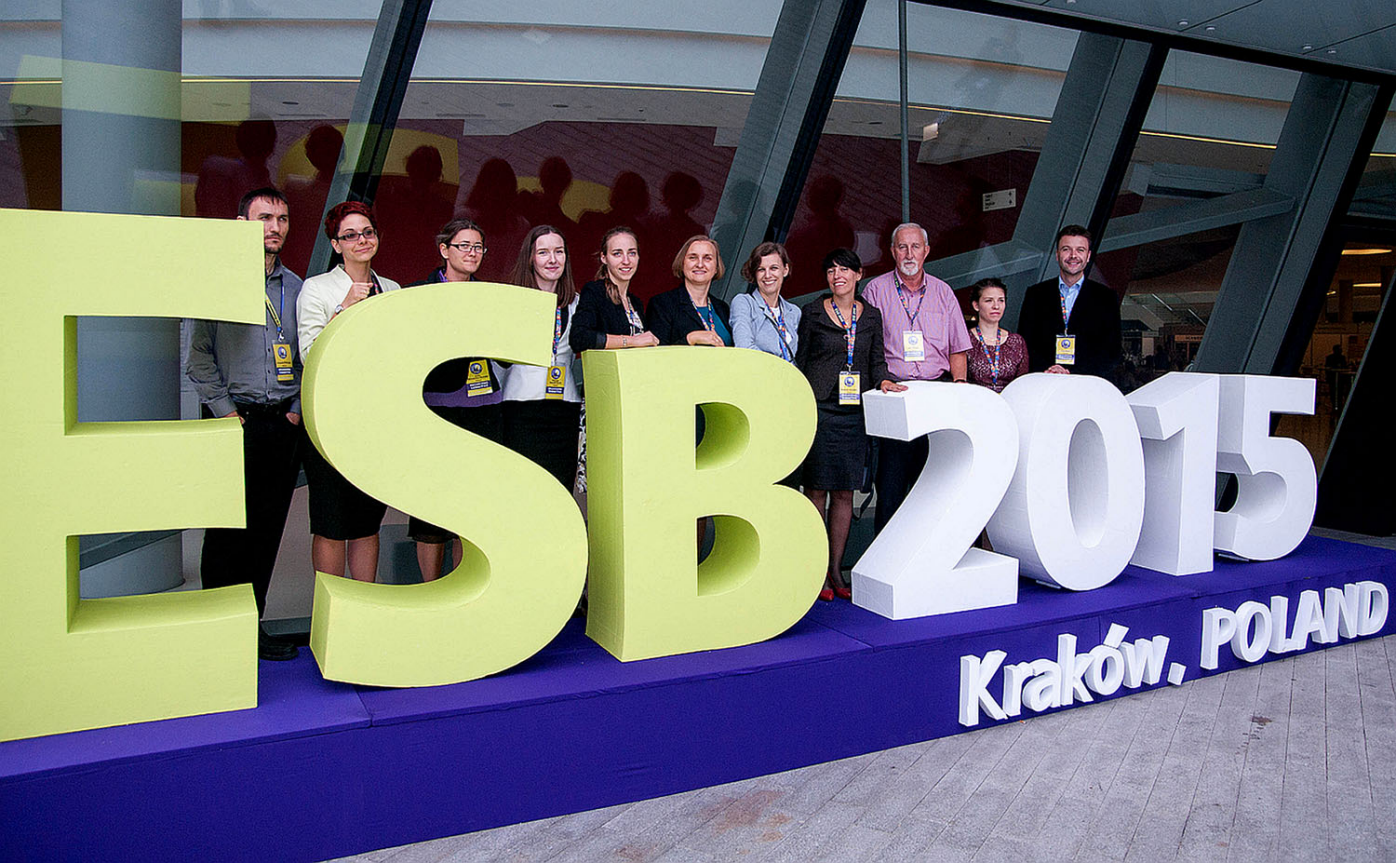
ESB 2015 Local Organizing Committee (from *left* to *right*): Krzysztof Pietryga, Małgorzata Krok-Borkowicz, Anna Morawska-Chochół, Patrycja Domalik-Pyzik, Katarzyna Reczyńska, Elżbieta Pamuła, Katarzyna Trała, Barbara Szaraniec, Jan Chłopek, Łucja Rumian and Karol Gryń

On behalf of the whole Organizing Committee (Fig. 4) we would like to thank all participants, chairpersons, reviewers, members of the advisory committee, all organizations, institutions and companies for their invaluable help in the organization of the meeting. The ESB 2015 could not have been a success without their presence and willingness to share the research results and bold scientific ideas.

This ESB 2015 Special Issue of *Journal of Materials Science*: *Materials in Medicine* is a collection of top contributions suggested by the reviewers and presented during the conference. We would like to thank all the authors for the timely preparation and revision of the manuscripts for this Special Issue as well as to all reviewers for their valuable work. Finally we are grateful to Prof. Serena Best and Prof. Matteo Santin, the Editors of this journal, for their support and constructive advice.

